# PDGF inhibits BMP2-induced bone healing

**DOI:** 10.1038/s41536-023-00276-5

**Published:** 2023-01-11

**Authors:** Sanja Novak, Josip Madunic, Laura Shum, Milan Vucetic, Xi Wang, Hitoshi Tanigawa, Mallika Ghosh, Archana Sanjay, Ivo Kalajzic

**Affiliations:** 1 Center for Regenerative Medicine and Skeletal Development, UConn Health, Farmington, CT USA; 2 Biochemistry and Organic Analytical Chemistry Unit, Institute for Medical Research and Occupational Health, Zagreb, Croatia; 3 Center for Vascular Biology, UConn Health, Farmington, CT USA; 4 Department of Orthopeadic Surgery, UConn Health, Farmington, CT USA

**Keywords:** Experimental models of disease, Translational research

## Abstract

Bone regeneration depends on a pool of bone/cartilage stem/progenitor cells and signaling mechanisms regulating their differentiation. Using in vitro approach, we have shown that PDGF signaling through PDGFRβ inhibits BMP2-induced osteogenesis, and significantly attenuates expression of BMP2 target genes. We evaluated outcomes of treatment with two anabolic agents, PDGF and BMP2 using different bone healing models. Targeted deletion of PDGFRβ in αSMA osteoprogenitors, led to increased callus bone mass, resulting in improved biomechanical properties of fractures. In critical size bone defects BMP2 treatment increased proportion of osteoprogenitors, while the combined treatment of PDGF BB with BMP2 decreased progenitor number at the injury site. BMP2 treatment induced significant bone formation and increased number of osteoblasts, while in contrast combined treatment with PDGF BB decreased osteoblast numbers. This is in vivo study showing that PDGF inhibits BMP2-induced osteogenesis, but inhibiting PDGF signaling early in healing process does not improve BMP2-induced bone healing.

## Introduction

Bone fractures and small defects regenerate without scar formation. However, up to 10% of fractures do not heal properly, forming mal-union or nonunion which decreases patients’ quality of life and presents a significant socioeconomic problem^1^. In a critical size bone injury, bone tissue is not able to bridge the defect, and autologous bone graft or synthetic biomaterials with growth factors need to be applied within the defect to repair injury.

Periosteum is a thin connective tissue layer enriched in skeletal stem/progenitor cells (SSPCs), that can be identified by expression of skeletal muscle alpha actin (αSMA), a population shown to actively participate in the bone healing process^2–4^. During regeneration, SSPCs are recruited to the injury site, where they undergo rapid expansion, and under influence of growth factors and cell to cell contact differentiate into the osteochondrogenic lineage.

Bone morphogenetic proteins (BMPs) are important regulators of bone homeostasis and regeneration^5^. Infuse™ Bone Graft, containing rhBMP2 is FDA approved for spinal fusion in level 1 degenerative disk disease, open tibial fractures, alveolar ridge augmentation, and off label for bone defect healing^6–9^. Although BMPs have been extensively used, significant side effects, and necessity of supraphysiological dosage has precluded their wider use. Complexity of regulating BMP signaling involves various autoregulatory mechanisms that could be limiting its action. BMP inhibitors such as Noggin and Gremlin are induced by BMP2 treatment in osteoblastic cell lines and primary cells^10,11^. Besides, BMP signaling self-limiting its own action, other growth factors that are enriched within the injured bone tissue could also exert similar negative or positive effects affecting osteogenesis. Therefore, there is a need to better understand the mechanism controlling BMP2-induced osteogenesis to improve treatment options.

It has been shown that platelet-derived growth factor (PDGF) is a mitogenic factor, involved in maintaining an immature phenotype of periosteal SSPCs thereby controlling the maintenance of an osteoprogenitor pool, and promoting their chemotactic recruitment to the injury site^12^. PDGF is expressed by platelets, macrophages, osteoblasts, and fibroblasts and is present in callus tissue during all phases of bone healing^12–14^. PDGF increases bone formation in the healing of segmental defects when using osteoinductive equine bone and beta-tricalcium phosphate supplemented with PDGF in rats^15^, in periodontal defects^16–19^ and in human extraction sockets^20^. In vitro effect of PDGF on increased proliferation was confirmed on fibroblasts, osteoblasts, smooth muscle cells, and periosteal cells as well^14,21–24^. However, in vitro studies show an inhibitory effect of PDGF on mesenchymal stem cell osteogenesis^25,26^. Despite unclear cellular mechanisms of PDGF on the osteoprogenitor lineage, PDGF has been FDA approved for surgical fusion of ankle (tibiotalar joint) and/or hindfoot (including subtalar, talonavicular, and calcaneocuboid joints) and periodontal defects as an anabolic agent^27–30^.

Used alone, BMP2 or PDGF are osteoanabolics and improve bone regeneration^31^. The action of PDGF BB in periosteum is mediated via PDGF receptor α or β. Our previous results showed that the proportion of PDGFRα^−^β^+^ periosteal cells within the fracture increases during the early phase of fracture healing. Further, periosteal cell cultures treated with PDGF BB inhibited BMP2-induced osteogenesis by attenuating Smad1/5/8 phosphorylation^23^.

To the best of our knowledge, interaction of PDGF and BMP2 signaling in regulating osteogenesis during bone healing in a physiological setting has not been investigated. In the present study, we have used αSMACreER2 murine model to evaluate how deletion of PDGFRβ in osteoprogenitor cells affects femur fracture healing. Using a critical size bone defect, we evaluated the osteoanabolic effects of PDGF BB and BMP2 and the effect of PDGFR inhibition on BMP2-induced osteogenesis. This study shows negative impact of two osteoanabolic agents on bone healing when applied together in injury models.

## Results

### Mesenchymal progenitor cells express PDGFRβ during fracture healing

**Fig. 1:**
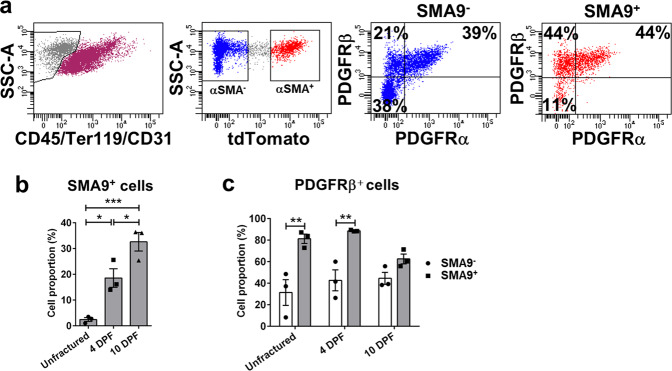
SMA9-labeled mesenchymal progenitor cells express PDGFRβ during fracture healing. Tibia fractures were created in 8 to 10-week-old SMA9 mice. Tamoxifen was injected on −1 and 0 DPF, and samples were collected for flow cytometry on 0 (unfractured periosteum), 4 and 10 DPF (fractured tibias). Two intact or fractured tibias were pooled for each sample, *n* = 3 for each group. **a** Representative dot plots for SMA9 cells and PDGFRβ^+^ cells gating in periosteal callus 4 DPF by flow cytometry. **b** SMA9 and **c** PDGFRs expression was analyzed within live, non-hematopoietic (CD45/Ter119/CD31)^−^ cells. Values are expressed as mean ± s.e.m. *p < 0.05, **p < 0.01, ***p < 0.001. Statistical test: One-way ANOVA with Tukey’s post hoc test (**b**) and two-way ANOVA with Tukey’s post hoc test (**c**).

To evaluate the expression of PDGFRβ in periosteal SSPCs, we utilized inducible αSMACreERT2 mice crossed with Ai9 reporter (SMA9). Population of SMA9 progenitors was evaluated in non-endothelial and non-hematopoietic cells (CD45/Ter119/CD31)^−^. Proportion of SMA9 cells in periosteal callus was significantly increased compared to unfractured control population (Fig. 1a, b). PDGFRβ expression was significantly increased in SMA9^+^ compared to SMA9^−^ population in unfractured and 4 days post fracture (DPF), but as SMA9 labeled osteoprogenitors undergo osteogenesis, statistical significance between SMA9^+^ and SMA9^−^ population expressing PDGFRβ was diminished (Fig. 1c).

### PDGFRβ deletion in osteoprogenitor cells improves fracture healing

**Fig. 2:**
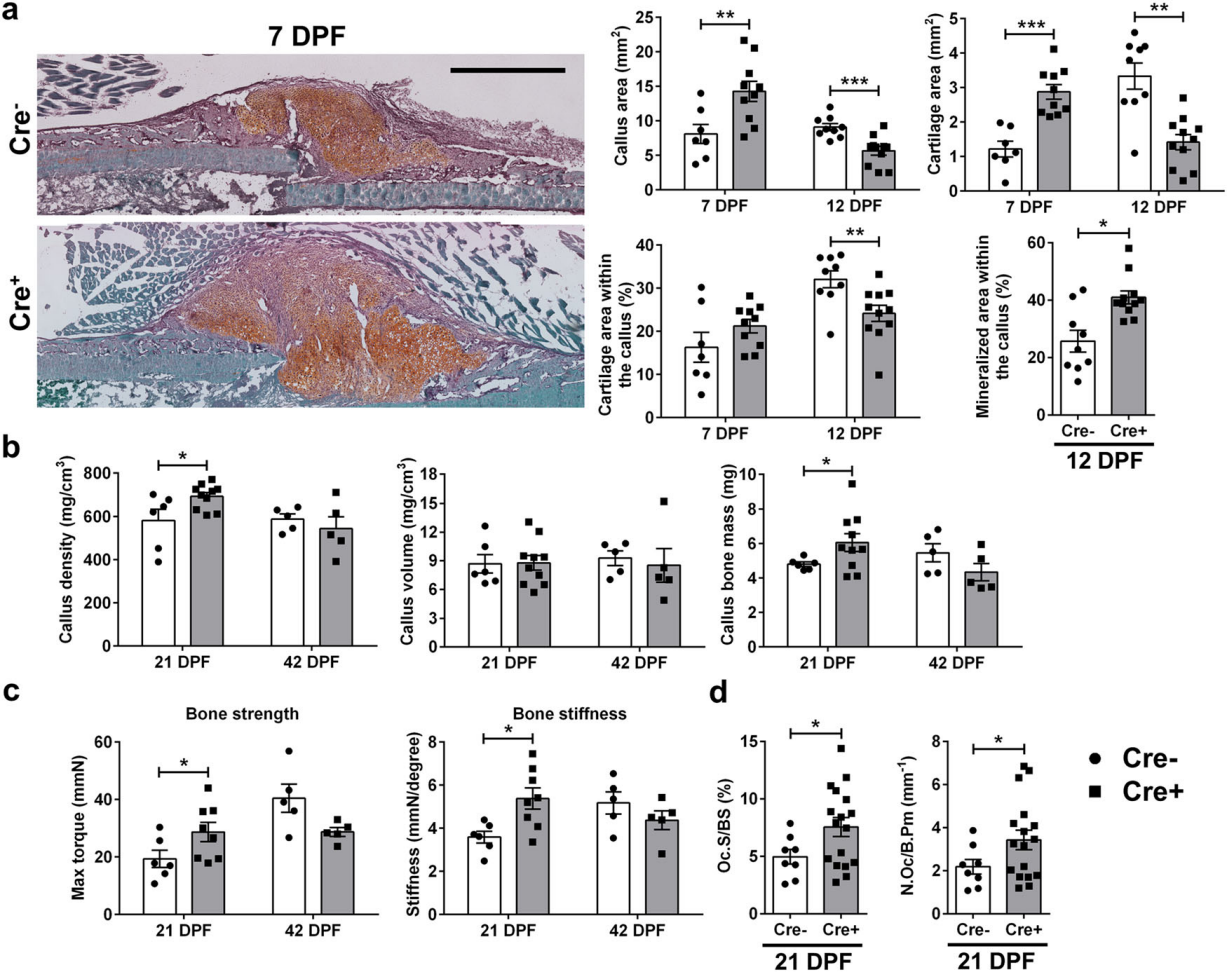
Deletion of PDGFRβ^fl/fl^ in progenitor cells improves fracture healing. Deleting of PDGFRβ in αSMA osteoprogenitors was induced by injecting tamoxifen on the day of femur fracture and 2 DPF. **a** Representative safranin O images of Cre^−^ and Cre^+^ fractured femurs 7 DPF. Increased callus size with increased cartilage area is present in Cre^+^ animals. Safranin O staining sections were analyzed on 7 and 12 DPF and von Kossa staining on 12 DPF. ImageJ was used to evaluate callus, cartilage and mineralized area. Cre^−^ *n* = 7, Cre^+^ *n* = 10 for day 7, and Cre^−^ *n* = 9, Cre^+^ *n* = 11 for day 12. Scale bar 1 mm. **b** PDGFRβ deletion in αSMA osteoprogenitors leads to changes in callus bone mass (Cre^−^ *n* = 6, Cre^+^ *n* = 10) and (**c**) stiffness 21 DPF (Cre^−^ *n* = 6, Cre^+^ *n* = 8). At 42 DPF there are no differences in structural or biomechanical femur properties (Cre^−^ *n* = 5, Cre^+^ *n* = 5). **d** Oc.S/BS and N.Oc/B.Pm are increased 21 DPF in Cre^+^ healing femurs (Cre^−^ *n* = 8, Cre^+^ *n* = 17). Values are expressed as mean ± s.e.m. *p < 0.05, **p < 0.01 ***p < 0.001. Statistical test: an unpaired two-tailed Students *t*-test. Oc.S/BS osteoclast surface per bone surface, N.Oc/B.Pm osteoclast number per bone perimeter.

To examine the effect of PDGFRβ deletion on fracture healing, we utilized an inducible αSMACreERT2 mouse crossed with PDGFRβ^fl/fl^ to generate αSMACreERT2/PDGFRβ^fl/fl^ compound mice (Supplementary Fig. 1a). Cre^−^ tamoxifen treated animals were used as controls. To induce PDGFRβ deletion, tamoxifen was injected on day 0 and 2 of fracture. PDGFRβ allele recombination was confirmed by PCR in the callus tissue, and in cells isolated from periosteal callus tissue at 5 DPF using flow cytometric analysis (Supplementary Fig. 1b, c). Deletion of PDGFRβ did not result in an increase in proportion of PDGFRα expressing cells (Supplementary Fig. 1d). We aimed to evaluate the in vivo effect of PDGFRβ deletion in αSMA osteoprogenitors during the fracture healing process using male mice. PDGFRβ deletion in αSMA cells resulted in a significantly increased callus area (*p* = 0.0097) and cartilage area (*p* < 0.001) 7 DPF (Fig. 2a). By 12 DPF cartilage area within the Cre^+^ callus was significantly lower than in Cre^−^ controls (*p* = 0.005) and the group with the PDGFRβ deletion showed significantly more mineralized area within the callus (~35% more than in Cre^−^, *p* = 0.012). PDGFRβ deficiency at 21 DPF resulted in increased callus bone mass, and fractured bones were stronger with increased bone stiffness compared to their Cre^−^ littermate controls (*p* < 0.05) (Fig. 2b, c). At 21 DPF, there was an increase in the number of osteoclast and osteoclast surface per bone surface in the remodeling callus tissue of Cre^+^ animals compared to Cre^−^ animals (Fig. 2d). We did not observe statistical differences in bone properties 42 DPF suggesting that PDGFRβ deletion in αSMA osteoprogenitors cells does not lead to excessive bone formation and abnormal healing phenotype (Fig. 2b, c). To determine if PDGFRβ deletion is affecting endogenous BMP downstream signaling, we performed histological analysis of Osterix and ALP staining of fractured femur on 7 DPF. We did not observe difference in Osterix (Cre- 1266 ± 206 cells/mm^2^, Cre+ 831 ± 167 cells/mm^2^, *p* = 0.133) and ALP (Cre- 32.19 ± 2.87% area, Cre+ 28.34 ± 2.83% area, *p* = 0.377) staining within the periosteal callus, indicating that PDGFRβ deletion is not affecting BMP signaling at that time point. This fracture study suggests that there is accelerated bone healing in animals with PDGFRβ deletion in αSMA osteoprogenitors cells. PDGF-mediated signaling controlled osteogenesis during the healing process. In contrast to data on male mice, fracture healing in female mice did not show significant changes between experimental groups (Supplementary Fig. 2).

### Inhibitory effect of PDGF BB on BMP2-induced osteogenesis

**Fig. 3:**
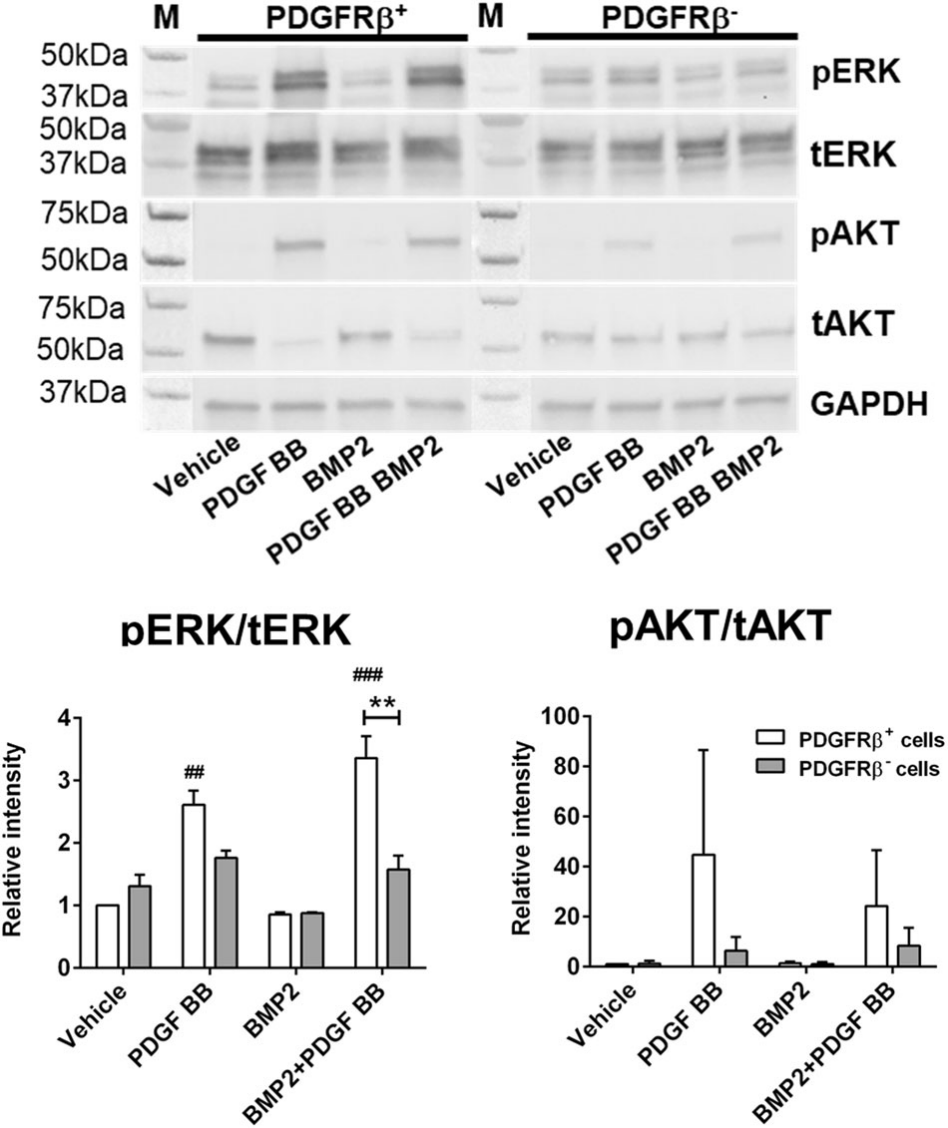
Deletion of PDGFRβ blocks ERK1/2 signaling upon PDGF BB treatment. Cultured periosteal cells from PDGFRβ^fl/fl^ mice, transduced with Ad-GFP or Ad-Cre. Ad-GFP were sorted for PDGFRβ^+^ cells and PDGFRβ^−^ cells were sorted from Ad-Cre transduced cells. Cell lysates were immunoblotted for pERK1/2, tERK, pAKT, tAKT or GAPDH. Values are expressed as mean + s.e.m. *n* = 2. **p < 0.001, ^#^*p* < 0.05, ^##^*p* < 0.001 different from Ad-GFP vehicle. M – protein ladder. Colorimetric image is stitched to original blot to visualize protein size. Statistical test: Two-way ANOVA with Tukey’s post hoc test.

We showed that PDGF BB inhibited in vitro osteogenic differentiation induced by BMP2 (Supplementary Fig. 3)^23^. To further examine the role of PDGFRβ signaling in periosteal cells, we evaluated effect of conditional deletion of PDGFRβ^fl/fl^_._ Periosteal cells were transduced with adenovirus carrying CMV-Cre (Ad-Cre) to induce PDGFRβ deletion and adeno-GFP (Ad-GFP) used as a control vector. To investigate role of PDGFRβ upon PDGF/BMP2 treatment we evaluated PDGF downstream signaling in sorted cells in which PDGFRβ was deleted by Ad-Cre (PDGFRβ^−^ cells) and cells transduced with Ad-GFP and sorted as PDGFRβ^+^ cells (Supplementary Fig. 4a). PDGF BB treatment (alone or in combination with BMP2) induced significant phosphorylation of ERK1/2, while BMP2 alone had no effect on its phosphorylation (Fig. 3, Supplementary Fig. 4b) in PDGFRβ^+^ cells. In PDGFRβ^−^ cells, PDGF BB did not induced significant phosphorylation of ERK1/2 nor AKT. Combined PDGF BB and BMP2 treatment in PDGFRβ^+^ cells lead to significant increase in pERK1/2 compared to PDGFRβ^−^ cells, indicating that phosphorylation of ERK upon PDGF BB treatment is dependent of PDGFRβ activation. pAKT in PDGFRβ^−^ cells with PDGF BB treatment was decreased, but did not reach statistical significance, possibly due to PDGFRα activation (Fig. 3).

**Fig. 4:**
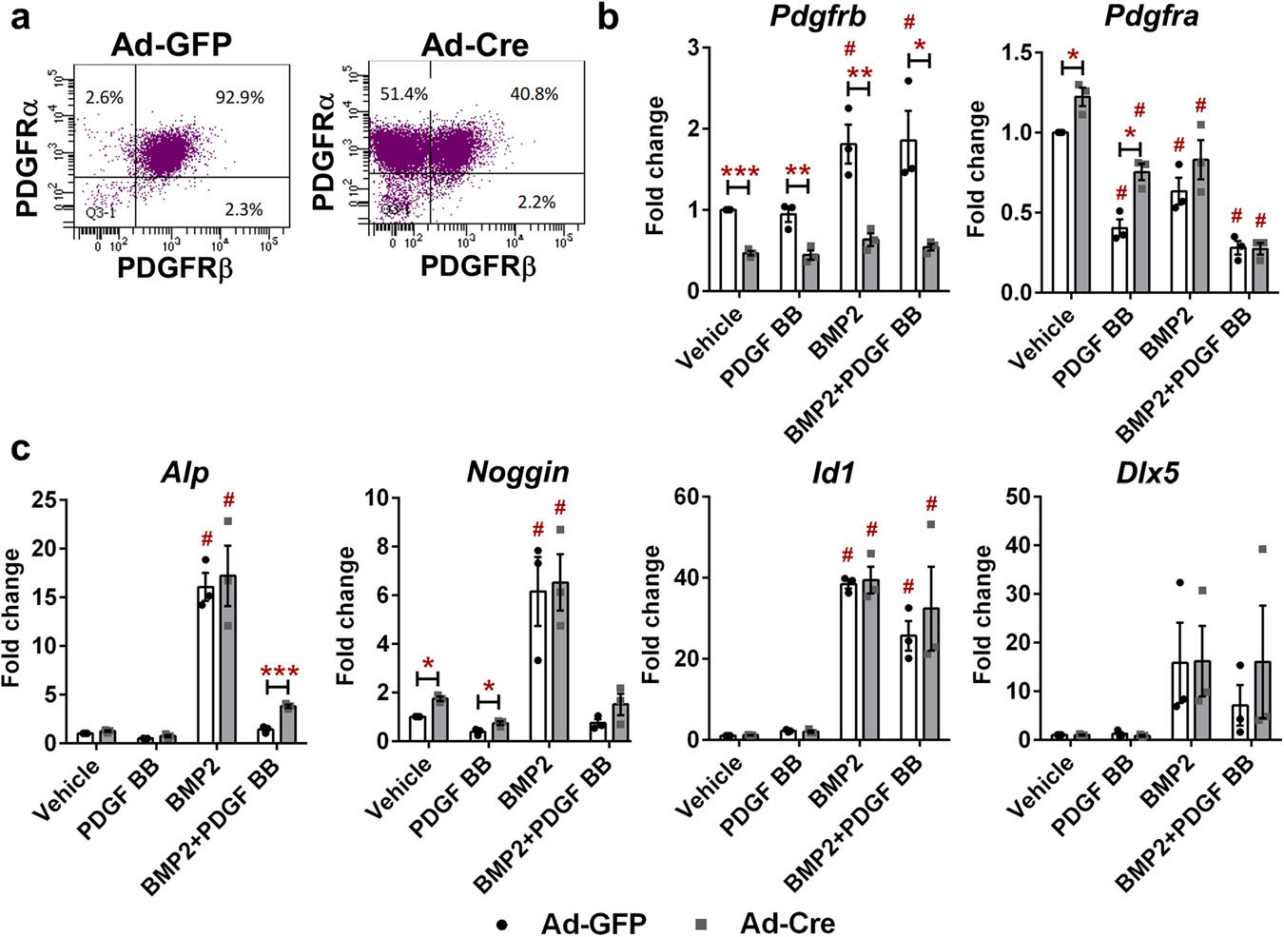
Effect of PDGFRβ deletion in periosteal cells on BMP2 downstream signaling. Ad-Cre deletion of PDGFRβ showed decrease in expression at (**a**) protein and (**b**) mRNA level. **c** BMP2 induced expression of *Alp*, *Noggin, Id1 and Dlx5*, while PDGF BB treatment blocked their upregulation in combined PPDGF BB + BMP2 treatment. Deletion of PDGFRβ using Ad-Cre partially restored *Alp* expression upon combined BMP2 + PDGF BB treatment. *n* = 3. Values are expressed as mean ± s.e.m. *p < 0.05, **p < 0.01, ***p < 0.001, # different from its vehicle treatment control. Statistical test: Two-way ANOVA with Tukey’s post hoc test.

We detected a decreased cell proportion of PDGFRβ^+^ cells in Ad-Cre compared to Ad-GFP cultures (95% in Ad-GFP to 40% in Ad-Cre), in cells before growth factor treatment, but no difference in cell proportion expressing PDGFRα (Fig. 4a). mRNA analysis after 24 h growth factor treatment, confirmed a decrease of ~60% in *Pdgfrb* gene expression in Ad-Cre compared to Ad-GFP cultures (Fig. 4b). BMP2 treatment of Ad-GFP transduced cells induced expression of *Pdgfrb*; whereas PDGF BB did not lead to *Pdgfrb* downregulation in combined PDGF and BMP2 treatment. *Pdgfra* expression in both Ad-GFP and Ad-Cre cell cultures upon growth factor treatment was significantly decreased in all treatment groups compared to their vehicle controls. However, PDGF BB treatment in Ad-Cre group led to increased expression of *Pdgfra* compared to Ad-GFP cultures (*p* = 0.027). BMP2 induced expression of *Alp*, *Noggin, Id1* and *Dlx5* as previously reported in ref. ^23^. PDGF BB treatment blocked BMP2-induced gene expression (Fig. 4c). Due to partial PDGFRβ deletion (60% cells), and intact PDGFRα signaling, only *Alp* expression was partially restored in Ad-Cre cells with combined PDGF BB + BMP2 treatment compared to Ad-GFP cells (*p* < 0.05).

### PDGF inhibits BMP2-induced osteogenesis during femoral defect healing

**Fig. 5:**
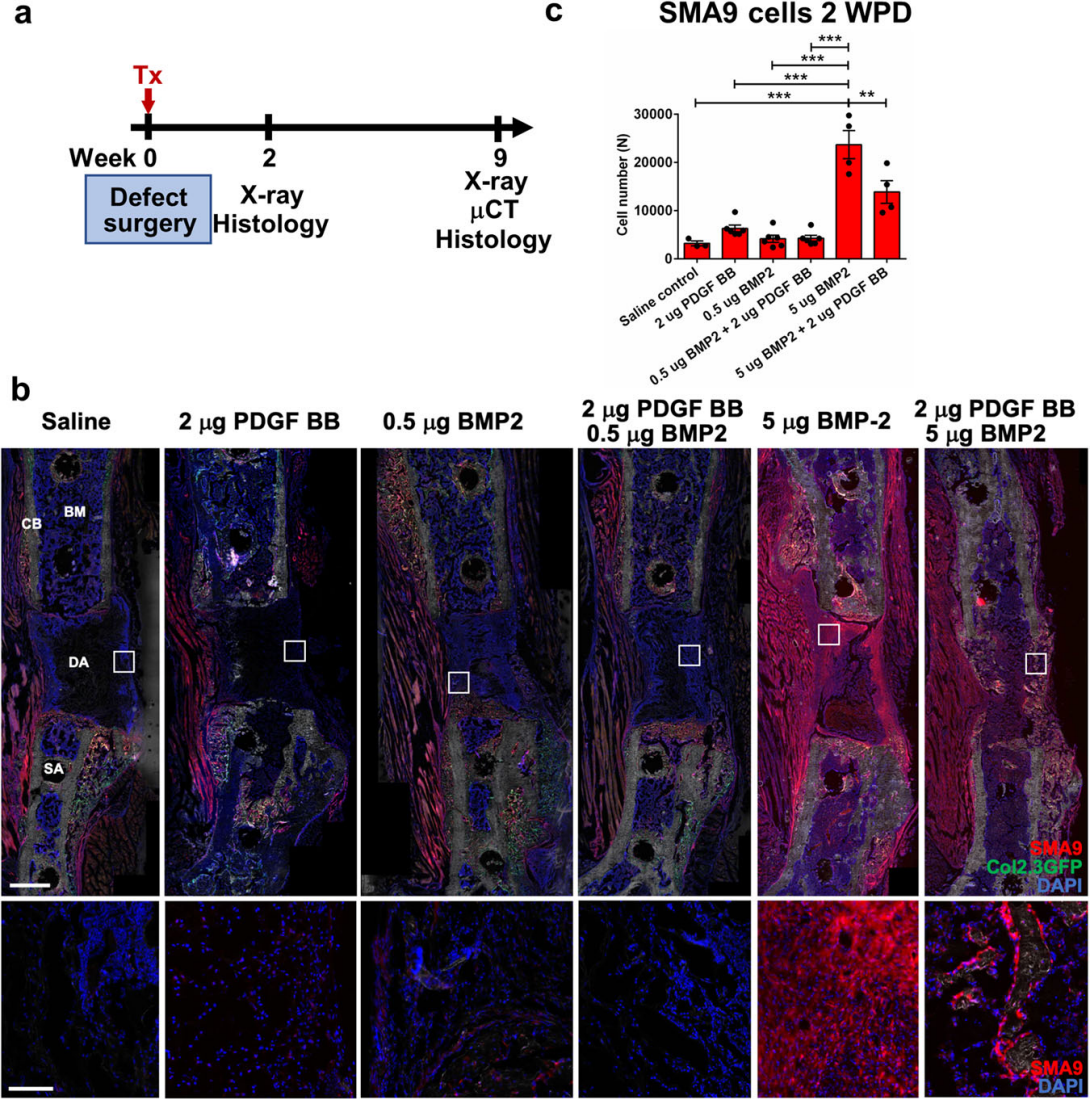
PDGF BB inhibits SMA9 cell expansion induced by BMP2 during femoral defect healing. **a** Experimental design. Femoral defects were performed on SMA9/Col2.3GFP male mice. Tamoxifen was administrated on the day of surgery and treatment combination were delivered in adsorbable collagen scaffold. X-ray was taken once per week to monitor defect healing process. **b** Representative images of the femoral defect 2 WPD surgery. Boxes demarcate magnified images to show SMA9^+^ and SMA9^−^ cells within the healing defect. Scale bar of defect images is 1 mm, and magnified image 250 μm. *n* = 3–6. **c** SMA9^+^ progenitor cells were assessed 2-weeks post defect (WPD) surgery in the defect area ± 30% of the defect to capture periosteal and bone marrow response to healing process. All results are expressed as mean ± s.e.m. **p < 0.01, ***p < 0.001. Statistical test: One-way ANOVA with Tukey’s post hoc test.

Presented data on the fracture healing of αSMACreERT2/PDGFRβ^fl/fl^ animals indicated an inhibitory role of PDGF signaling on osteogenesis. We aimed to determine the role of PDGF signaling on the clinically relevant healing model of BMP2-induced osteogenesis. We used a 3 mm critical size femoral defect healing in triple transgenic αSMACreERT2/Ai9/Col2.3GFP mice (termed SMA9/Col2.3GFP). We were able to evaluate the effects on osteoprogenitor (SMA9^+^ cells), and on mature osteoblast (Col2.3GFP^+^ cells) contribution to critical size femoral defect healing. Tamoxifen was administrated on the day of surgery and SMA9^+^ progenitor cells were assessed 2 weeks post defect (WPD) surgery. Histological analysis showed that lower dose of BMP2 (0.5 µg) and PDGF BB (2 µg) did not induce great cell migration into the scaffold nor significant SMA9^+^ cell expansion within the absorbable collagen sponge (Fig. 5 and Supplementary Fig. 5a). SMA9 cells were primarily located on the outer layers of the scaffold (Fig. 5b). A higher dose of BMP2 (5 µg BMP2) induced migration of osteoprogenitor cells deep into the scaffold, as evidenced by increased number of SMA9^+^ cells within the defect compared to all other single treatment groups (*p* < 0.001). We also confirmed that the combined 5 μg BMP2 + 2 μg PDGF BB treatment resulted in a significant decrease of SMA9^+^ cells when compared to 5 µg BMP2 treatment alone (*p* = 0.002) (Fig. 5b, c).

**Fig. 6:**
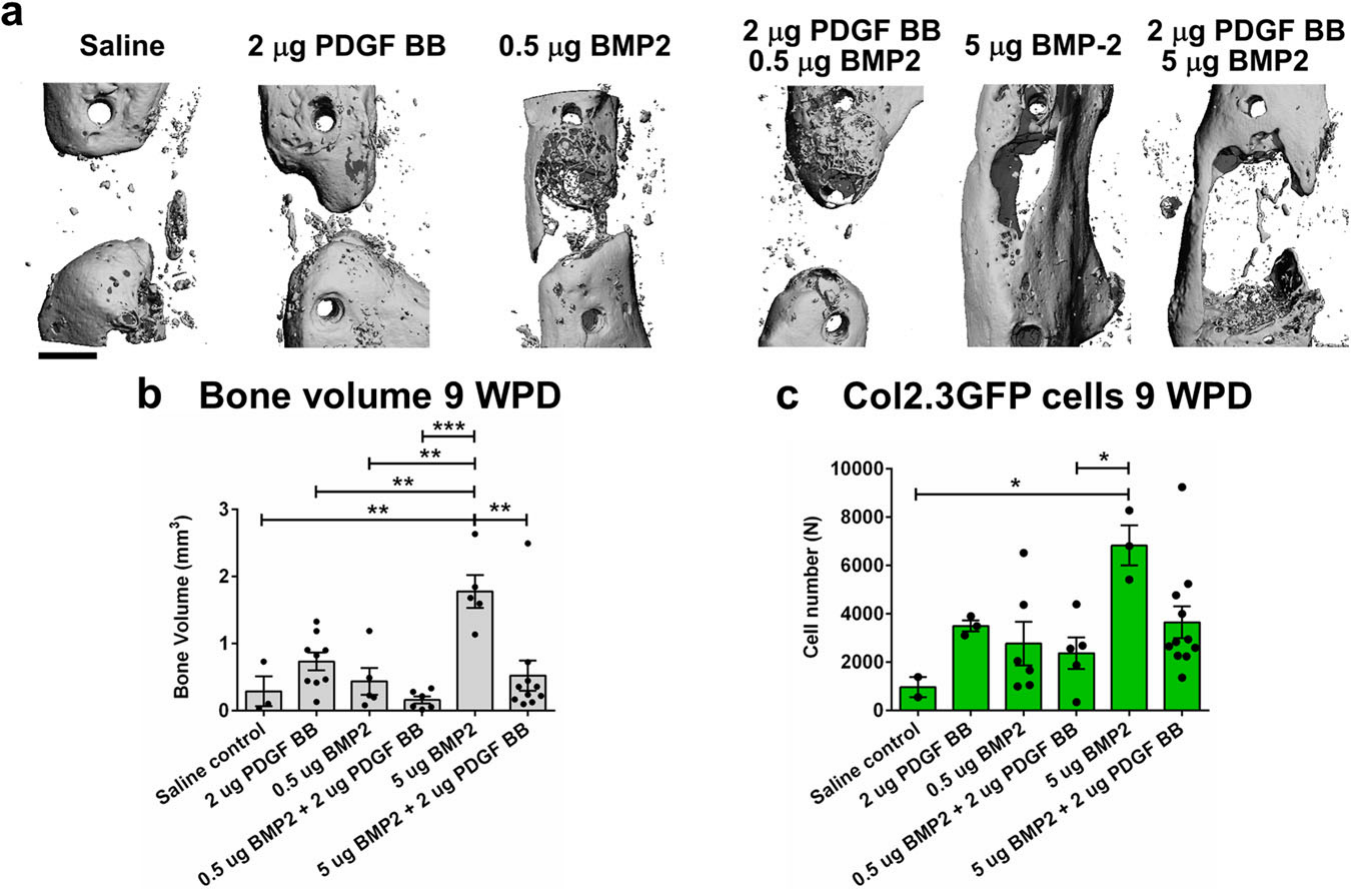
PDGF BB inhibits BMP2-induced osteogenesis in critical femoral defects. **a** Representative 3D images of μCT morphometry of healing defect 9 WPD with bone bridging. **b** Evaluation of bone volume within the defect with highest bone volume in group treated with 5 µg BMP2. **c** Histological evaluation of Col2.3GFP^+^ cells within defect area 9 WPD surgery. Scale bar of defect images is 1 mm. Saline control *n* = 2, *n* = 3–10 mice per experimental group. All the results are expressed as mean ± s.e.m. *p < 0.05, **p < 0.01, ***p < 0.001. Statistical test: One-way ANOVA with Tukey’s post hoc test.

Nine weeks post defect surgery, μCT analysis showed 2 μg PDGF BB and 0.5 μg BMP2 treatment induced modest bone formation within the defect (Fig. 6a). When the low dose of BMP2 (0.5 μg) was combined with PDGF BB bone formation was comparable to saline control (Fig. 6a, b). The higher dose of BMP2 (5 μg) induced significant bone formation and bridging, and PDGF BB inhibited BMP2-induced bone formation (5 μg BMP2 compared to 5 μg BMP2 + 2 μg PDGF BB, *p* = 0.001) (Fig. 6a, b). Histological evaluation at 9 WPD showed an increase in Col2.3GFP^+^ osteoblasts within the defect area in BMP2 treated group (5 μg) and combined BMP2 and PDGF BB treatments were not different then saline control (Fig. 6c). SMA9^+^Col2.3GFP^+^ osteoblasts are increased in 5 μg BMP2 treated group compared to saline control group, while combined PDGF BB + BMP2 treatment decreased SMA9^+^Col2.3GFP^+^ osteoblast number (statistically not significant) (Supplementary Fig. 5b). A population of Col2.3GFP osteoblasts that is not derived from SMA9^+^ cells does not show effects of PFGF BB or BMP2 treatment (Supplementary Fig. 5c).

**Fig. 7:**
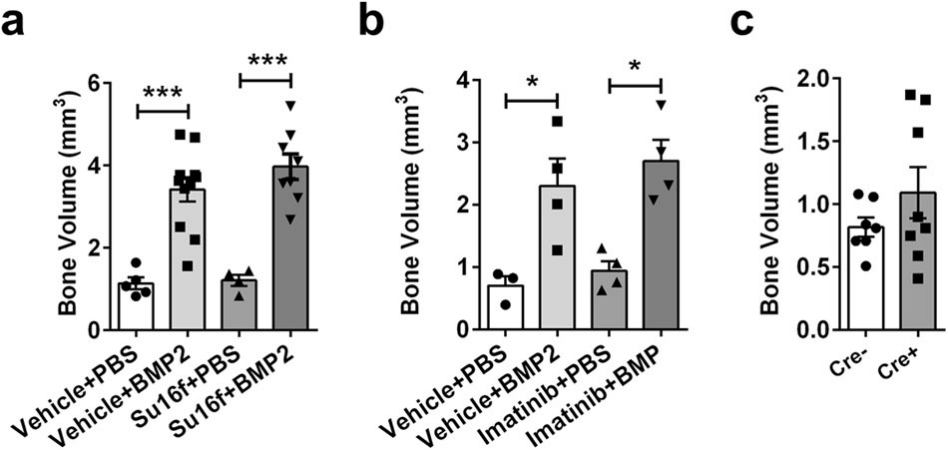
PDGFRβ inhibition does not positively affect critical femoral defect healing. **a** Femoral defects performed on C57BL/6J male mice treated with 10 mg/ml Su16f or vehicle in a period of two days before defect surgery till two days post, by oral gavage. Adsorbable collagen sponge was loaded with 5 μg of BMP2 or PBS on a day of surgery. Femurs were evaluated by μCT 9 WPD (Vehicle + PBS *n* = 5, Vehicle + BMP2 *n* = 11, Su16f + PBS *n* = 4, Su16f + BMP2 *n* = 8). **b** Imatinib (50 mg/kg/day, ip) was used to inhibit PDGFRβ from two days before surgery till 2 days post-surgery, femurs were evaluated by μCT 9 WPD (Vehicle + PBS *n* = 3, Vehicle + BMP2 *n* = 4, Imatinib + PBS *n* = 4, Imatinib + BMP2 *n* = 4). **c** PDGFRβ deletion did not affect critical size femoral defect healing in αSMACrePDGFRβ^fl/fl^ animals (tamoxifen induced deletion at −3/0/3 days post defect, Cre- *n* = 7, Cre+ *n* = 8). All the results are expressed as mean ± s.e.m. *p < 0.05, ***p < 0.001. Statistical test: One-way ANOVA with Tukey’s post hoc test.

To determine potential translational impact of inhibiting PDGFRβ in vivo, we have used two different approaches using two different PDGFR inhibitors, i) specific PDGFRβ inhibitor – Su16f (10 mg/kg/day) and ii) tyrosine kinase inhibitor Imatinib (50 mg/kg/day). Animals were treated with inhibitors or its vehicle in period of -2 till +2 days of femoral defect surgery with scaffolds loaded with BMP2 or PBS as a control. Without BMP2 loaded on a scaffold, there is minimal bone formation within the defect determined by μCT analysis 9 WPD. However, no additional effect of PDGFR inhibitors (Su16f Fig. 7a, and Imatinib Fig. 7b) on femoral defect healing was observed.

Next, we evaluated whether the deletion of PDGFRβ in osteoprogenitors affects healing when exogenously adding BMP2. We evaluated critical size defect healing in αSMACreERT2/PDGFRβ^fl/fl^ animals in which PDGFRβ deletion was induced by tamoxifen on -3/0/3 days of defect. The effective dose of BMP2 (5 μg) was loaded on the scaffold during femoral defect surgery. We did not detect difference in bone volume within the defect 9 WPD. Along with series of experiments in which we used PDGFRβ inhibition, this result shows that inhibiting endogenous PDGFRβ signaling does not affect BMP2 healing response during early time points of healing process (Fig. 7c).

## Discussion

Despite number of studies evaluating the effects of growth factors on bone regeneration the lack of efficient bone healing precludes direct clinical application.

In the present study we show high expression of PDGFRβ in the periosteum and periosteal callus during early fracture healing. αSMA osteoprogenitor cells have significantly higher expression of PDGFRβ compared to other non-hematopoietic, non-endothelial cells (CD45/Ter119/CD31)^−^.

We evaluated in vivo effects of PDGFRβ deletion in αSMA osteoprogenitors in a stabilized fracture healing model using αSMACreERT2/PDGFRβ^fl/fl^ mice. αSMACre has been shown to identify osteochondroprogenitors^3,4^. Callus size and cartilage area were increased 7 DPF in male mice with PDGFRβ deletion compared to their Cre^−^ controls. Cartilage formation is important for initial bridging, increases callus size, and provides more stabilization to a fractured bone^32^. Intact PDGF signaling in endothelial cells and its effect on neovascularization could regulate osteogenesis later in healing phase. Deletion of PDGF BB in TRAP positive cells using Trap-Cre mice leads to lowering of vessel volume and surface area, and decreased proliferation of endothelial cells which consequently lowered osteocalcin positive osteoblast cell numbers on trabecular and periosteal bone^33^. With use of targeted PDGFRβ deletion in αSMA osteoprogenitors, PDGFRβ signaling in other cell lineages remains intact, providing normal signals for angiogenesis^33–35^. Deletion of PDGFRβ in osteoprogenitors did not have an effect on Osterix and ALP staining at 7 DPF possibly indicating normal BMP signaling at that time point in healing process. On day 12 post fracture, cartilaginous area within the Cre^+^ animals was decreased, and replaced by the woven bone with a significantly increased mineralized area in the callus. By day 21 DPF mice with αSMACre directed PDGFRβ deletion had significantly increased callus bone mass and biomechanical properties (strength and stiffness). Likewise, systemically expressing Cre targeted PDGFRβ deletion in 4-week-old mice increased the woven bone to callus ratio^25^. Bohm et al. showed increased tibia callus BV/TV and BMD when PDGFRβ was deleted in Osx^+^ cells which would indicate improvement in healing at 14 DPF^12^. However, the authors observed impaired callus formation at earlier time points with signs of delayed healing. Two aspects of this study; use of the constitutively active Osx-Cre and a mixed background of the mice may confound the results. Our and other data show that deletion of PDGF signaling in osteogenic lineage results in improved bone formation during regeneration^36^.

In female αSMACreERT2/PDGFRβ^fl/fl^ mice we did not observe any differences in a healing callus between Cre^+^ and Cre^−^ mice (Supplementary Fig. 2). Youngstrom et al. show that *Acta2* (SMA) expression in male mice is higher compared to female mice^37^ which might lead to observed differential phenotype in male and female animals seen in our studies. Furthermore, compared to males, female C57BL/6 mice at 3 and 6 months of age have lower bone volume, bone marrow stromal cells derived from female mice have lower osteoblastic differentiation potential, and form less CFUs with lower ALP frequency^38^. Differences within osteoprogenitor pool in bone marrow stromal cells contribute to osteoblastic differentiation potential, possibly leading to sexual dimorphism and lack of healing phenotype in female mice in our studies.

We have previously demonstrated the inhibitory effect of PDGF BB on BMP2-induced osteogenesis of periosteal cultures which was mediated through inhibition of canonical BMP2/Smad signaling pathway^23^. This effect is attributable to PDGFRβ directly, because chemically inhibiting PDGFRβ signaling with Su16f rescued the effect of PDGF on BMP2-induced osteogenesis^23^. Most of the αSMA cells co-express PDGFRα and PDGFRβ (Fig. 1a). In the present study we show that deletion of PDGFRβ significantly induced *Alp*, *Noggin*, *Id1*, and *Dlx5* gene expression with BMP2 treatment, and partly rescued inhibited BMP2-induced *Alp* expression with PDGF BB treatment. This data shows inhibitory role of PDGF BB signaling on BMP2-induced osteogenesis. Complete inhibition of ERK1/2 phosphorylation with PDGFRβ deletion show importance of PDGFRβ signaling on ERK1/2 regulation of its downstream signaling. However, with deletion of PDGFRβ, full osteogenic potential with BMP2 treatment is not restored, as PDGFRα signaling, could have an effect on the BMP2 signaling through phosphorylation of AKT. Deletion of only one of the two PDGFRs in Osterix-positive cells does not affect bone phenotype, however, deletion of both isoforms of PDFGRs in osteoblast lineage cells increases trabecular bone volume in male and female indicating redundant role of PDGFRs^36^.

Reports of significant side effects of BMP2 noticed in patients during off label use, prompted us to evaluate inhibitory role of PDGF signaling on BMP2-induced bone formation. PDGF and BMP2 have the ability to induce bone regeneration, while significant inhibition of BMP2-induced bone formation was noticed with combined PDGF BB treatment in vitro. We investigated these effect on in vivo healing of critical size long bone defects, a clinically relevant healing model. Pharmacokinetic studies in nonhuman primates and in rats showed rapid systemic clearance of BMP2 after intravenous injection, with longer retention present when rhBMP2 is loaded on the absorbable collagen sponge^39^. Therefore, we utilized an adsorbable collagen sponge (Medtronic) to deliver growth factors. Treatment with 5 µg BMP2 significantly increased progenitor cell presence (SMA9^+^) within the defect. When the scaffold was loaded with 5 µg BMP2 and 2 µg of PDGF BB combined, significantly fewer number of SMA9 cells were present within the defect area as compared to BMP2 treatment alone. This SMA9 osteoprogenitors contributed to Col2.3GFP osteoblasts. Proportion of SMA9^+^Col2.3GFP^+^ at 9 WPD surgery was about 25% indicating presence of Col2.3GFP cells that are not derived from SMA9 progenitors. This data is consistent with our published results where ~25% Col2.3GFP^+^ osteoblasts were labeled by long term SMA9 osteoprogenitors in a fracture healing^3^.

It is important to mention that SMA9^+^ population labeled (tamoxifen treatment) at the time of defect procedure shows clear effect of BMP2 on this cell population. Other osteogenic populations that appear later during repair (not labeled with SMA9) are likely not exposed to the exogenously added PDGF or BMP2. This indicates the effect of BMP2 is short lived and critical for the early events of the healing, such as expansion and early commitment of SMA9 labeled population.

In addition, the proportion of SMA9 contribution can be lower due to 50–60% recombination efficiency of a SMACreER mice confirmed in vitro and in vivo using DTA ablation model^3,40^. αSMA identifies some, but not all cells contributing to osteoblasts and chondrocytes during bone healing. We have shown that Col2.3CreER-labeled cells contribute to osteoblast formation in the fracture callus^3^. Work by other groups showed Prx1, Pdgfra, Lepr, Cxcl12, Gli1, CatK, Mx1 cell contribution to callus formation, however, the overlapping proportion of those markers within the healing periosteum has not been confirmed^41^.

Our in vivo data emphasizes the negative role of PDGF signaling in regulating BMP2-induced osteogenesis when added exogenously. However, we did not observe positive effect on BMP2-induced osteogenesis in critical size femoral defect healing by inhibiting PDGF signaling in bone regeneration using PFGFR inhibitors or deleting PDGFRβ in osteoprogenitors. Exogenously added PDGF might potentially modulate inflammatory response and lead to observed negative effect on BMP2 signaling during regeneration^42^.

This study shows that two individually anabolic agents, when combined, exhibit negative effects on osteogenesis. With the mitogenic and chemotactic abilities of PDGFs on local injured tissue, and the osteogenic ability of BMP2, our data address the importance of temporal and spatial presence of PDGF and/or BMP2 for proper healing. Our study showed that inhibition of PDGF signaling early in the healing process does not lead to improved bone healing abilities. Future studies are needed to determine optimal treatments and ways to decrease the supraphysiological dose of BMP2 that is presently used to induce bone regeneration and diminish significant side effects in patients^43^.

## Methods

### Animal models

All of the animal procedures were approved by the UConn Health Institutional Animal Care and Use Committee, and experiments were performed in accordance with its guidelines and regulations, and authors complied with the ARRIVE guidelines. We used αSMACreERT2 and Col2.3GFP mice that have been previously described in refs. ^4^^,^^44^. Ai9 reporter mice (007909), were purchased from Jackson Labs^45^. We backcrossed PDGFRβ^fl/fl^ (129S4/SvJaeSor) generated by Dr. Phillipe Soriano^46^ for 8 generations to C57Bl/6J background. All strains were maintained on a C57Bl/6J background. Mice were housed in ventilated cages, maintained at 22 ± 2 °C, 55 ± 5% humidity, and 12-h light/dark cycle with ad libitum access to food and water. αSMACreERT2 mice were crossed with the Ai9 reporter mice (Jackson Labs) and Col2.3GFP mice, and triple transgenic male mice αSMACreERT2/Ai9/Col2.3GFP (termed SMA9/Col2.3GFP) were used at the age of 5–7 months for evaluation of growth factor efficiency. For fracture healing, αSMACreERT2/PDGFRβ^fl/fl^ Cre^+^ experimental animals and Cre^−^ littermates as controls at 8–10 weeks of age, were treated with tamoxifen (75 µg/g). We determined genotype for Cre, Pdgfrβ and DNA recombination by PCR (primer sequences in Supplementary Table 1).

### Materials

Collagenase P (Roche, IN, USA), hyaluronidase (Sigma Aldrich, St Louis, MO, USA), Ad-GFP (Vector Biolabs, Cat No. 1060), Ad-Cre (Vector Biolabs, Cat No. 1045), PDGF-BB (R&D Systems, Minneapolis, MN, USA), recombinant human BMP2 (PeproTech, Rocky Hill, NJ, USA), TRIzol reagent (Thermo Fisher Scientific, Waltham, MA, USA), ImProm II kit (Promega, USA), TaqMan Universal Mastermix (Thermo Fisher Scientific, UK), SYBR green-based assay (Thermo Fsher, Waltham, MA, USA; Life Technologies, Grand Island, NY, USA), Protease Inhibitor cocktail (Roche), tamoxifen (Sigma Aldrich, St. Louis, MO, USA), corn oil (Acros Organics, Belgium), Cryomatrix (Thermo Scientific, Waltham, MA, USA), Leukocyte acid phosphatase kit (Sigma Aldrich, St. Louis, MO, USA), BMP2 (Medtronic, MN, USA) and PDGF BB (R&D, 520-BB-050/CF), methyl methacrylate bone cement (Orthodontic Resin, Dentisply Caulk Inc. Milford, DE, USA). Antibodies used are listed in Supplementary Table 1.

### Flow cytometry

Periosteal callus cells were isolated as previously described in refs. ^47^^,48^. For evaluating PDGFRβ expression in αSMA cells, two intact or fractured tibias were pooled in one sample. Cells were washed, stained and resuspended in staining medium (2% FBS, 1 mM EDTA in PBS). CD45 Fluor 450, Ter119 eFluor 450 and CD31 eFluor 450 antibodies were used to exclude hematopoietic and endothelial cells from the periosteal population. CD140a PE-Cy7 or APC and CD140b APC or eFluor780 antibodies were used to determine PDGFRα and PDGFRβ expression level in SMA9 cells. Before acquiring, cells were stained with DAPI (50 ng/ml final concentration) for dead cell exclusion. Single cell controls, fluorescence minus one (FMO) and isotype controls were used for setting the gates. LSR II flow cytometry system (BD Bioscience, San Jose, CA) was used for sample acquisition. Voltages and gates were set based on unstained and single stained and fluorescence minus one (FMO) controls. Diva 8 software (BD Bioscience) was used for data analysis.

### Bone fractures

To evaluate PDGFRβ and PDGFRα expression during bone healing, tibia fracture model in SMA9/Col2.3GFP mice was used as previously described in ref. ^3^. Animals were injected with 75 µg/g of tamoxifen the day before and on the day of tibial fracture to induce tdtomato expression. Animals were sacrificed, and samples collected from the unfractured leg, and 4- and 10-days post fracture (DPF) for flow cytometry analysis.

Femoral fractures were performed in 8–10-week-old animals as previously described in ref. ^47^. Fractures were confirmed immediately after the procedure and the fracture healing process was monitored by X-ray (Faxitron LX 60 and Parameter2D, Kubtec) at 7, 14, 21- and 42 DPF. To induce PDGFRβ deletion in the αSMA cells of a αSMACreERT2/Pdgfrβ^fl/fl^ mice, tamoxifen (75 µg/g, *ip*) on the day of fracture and 2 DPF was injected to both Cre^+^ and Cre^−^ groups. Buprenorphine (0.1 mg/kg) was given subcutaneously every 10–12 h for the first 2 days of healing. To reduce animal numbers, for evaluating PDGFRβ expression and cell recombination, both right and left tibia fractures were performed. Fracture procedure was the same as for femur fractures, only a 27 G needle for fracture stabilization was used.

### Histology

Femurs were fixed in 4% paraformaldehyde for 3 days at 4 °C, incubated in 30% sucrose/PBS overnight, intramedullary pins were removed, and the femurs were embedded in Cryomatrix. 7 μm cryosections were collected using a tape transfer system (Section-lab, Japan)^49^. Callus area, cartilage area within the callus and mineralization was determined by Safranin O and von Kossa staining as previously describe in ref. ^47^. TRAP staining was performed on 21-day fractures with Leukocyte acid phosphatase kit according to the manufacturer’s protocol and sections evaluated using OsteoMeasure software (OsteoMetrics Inc., Atlanta, GA, USA). A minimum of two sections were stained, scanned using Axioscan microscope (Zeiss, Germany) and analyzed with ImageJ software (NIH, USA). Osterix staining was perform as previously published in ref. ^3^. To detect alkaline phosphatase Vector® Blue Substrate Kit (SK-5300, Vector laboratories) was used with 7 min of incubation time.

### Micro computed tomography (µCT) and torsion testing for evaluating fractured callus

Three and six weeks after fracture, femurs were dissected, soaked in PBS wrapped gauze, and stored at −20 °C until µCT scanning. µCT40 (Scanco Medical AG, Bassersdorf, Switzerland) with a voxel size of 12 µm, 55 kV and intensity of 145 µA was used as described previously in ref. ^47^. Two hundred slices of middle fractured area were evaluated. Following µCT imaging, torsion testing was performed on the same samples. For torsion testing, samples were potted in methyl methacrylate bone cement and biomechanical properties were measured using the TestBench™ Torsion Testing system (Bose Corporation ElectroForce Systems Group, Eden Prairie, MN, USA) with data acquisition rate of 10 Hz, and 1 degree/s torsion as described previously in ref. ^47^.

### In vitro assay

Periosteal cells of PDGFRβ^fl/fl^ animals were isolated as previously described in ref. ^23^. Cells were seeded in αMEM/10% FBS and cultured for 4 days in a 37 °C humidified incubator, 5% O_2_, 5% CO_2_, on day 4 cultures were transferred to normal oxygen conditions. On day 5 cells were infected with 300 multiplicity of infection units (MOI) of Ad-GFP or Ad-Cre in αMEM 0.5% FBS for 24 h, washed and further processed for protein or RNA analysis.

### Immunoblot assay

For protein analysis, PDGFRβ^fl/fl^ cultures a day after Ad-GFP/Ad-Cre treatment were sorted to collect live, PDGFRβ^+^ cells from Ad-GFP treated cultures, and PDGFRβ^−^ cells from Ad-Cre cultures. To collect sufficient amount of proteins, cells were passaged two times, grown till reaching confluency, serum starved (0.1% BSA in DMEM) overnight, and treated with PDGF BB (10 ng/ml), recombinant human BMP2 (100 ng/ml), PDGF BB + BMP2 or vehicle (0.1% BSA) for 30 min. Cell lysates were harvested in 1% NP40 lysis buffer containing 1X complete Protease Inhibitor cocktail. Samples were separated by SDS-PAGE and transferred to nitrocellulose membrane, blocked in 1X TBST containing 5% dry milk, treated with primary Ab followed by secondary Ab and imaged by ChemiDoc Imaging system (Biorad, Hercules, CA, USA). tERK and tAKT were used for normalization of pERK and pAKT. To determine relative band intensity, unmodified blots were analyzed by ImageJ software (NIH, USA). Blots were cropped and colorimetric image with protein ladder stitched to original blot to visualize protein size. Blots presented are derived from the same experiment and were processed in parallel.

### Gene expression analysis

After viral transduction, cells were passaged to a 6-well plate at a seeding density of 2.5 × 10^5^ cells per well, and when confluent, cells were serum starved (0.1% BSA in DMEM) overnight, and then without previous sorting treated with PDGF BB (10 ng/ml), recombinant human BMP2 (100 ng/ml), PDGF BB + BMP2 or vehicle (0.1% BSA) for 24 h, after which cells were collected for RNA analysis. To confirm transduction efficiency prior growth factor treatment, PDGFRβ expression was evaluated by flow cytometry in transduced Ad-GFP and Ad-Cre cultures.

RNA was isolated from cultured periosteal cells using TRIzol reagent^44^. RNA concentration and purity were assessed on a NanoDrop One. Following DNase treatment, 1 µg of RNA, was synthesized into cDNA by ImProm II. TaqMan probes for *Alp* (Mm01187117_m1), *Ibsp* (Mm00492555_m1), *Bglap* (Mm03413826_mH), *Pdgfra* (Mm00440701) and *Gapdh* (Mm99999915_g1) with TaqMan Universal Mastermix were used to evaluate gene expression. *Pdgfrb, Noggin, Id1* and *Dxl5* expression were evaluated using a SYBR green-based assay, primer sequences in Supplementary Table 2. Data were normalized to *Gapdh* housekeeping gene, calculated using 2-ΔΔCt and presented as fold change compared to Ad-GFP control vehicle treated group.

### Critical size bone defect

Critical sized femoral defects (3 mm) were performed on 5 to 7-month-old male SMA9/Col2.3GFP using an established protocol^50^ and a commercially available external fixation (MouseExFix, RISystem). Males were used for critical size defect due of their bigger femur size making it more accessible for the external fixation placement. BMP2 (0.5 and 5 µg) and PDGF BB (2 µg) factors alone or in combination were applied to the defect in an adsorbable collagen sponge as a scaffold (Medtronic) cut to size (3 × 4 × 6 mm) on which growth factors were loaded in a 7 µl volume. To avoid infection, animals were treated with 1.5–4.5 mg/mouse/day sulfamethoxazole/trimethoprim in water two days before the surgery and for the initial two weeks after surgery. To induce tdtomato expression in αSMA cells (termed SMA9 cells), mice were treated with tamoxifen on the day of surgery. Animals were sacrificed at two time points, 2 weeks post-surgery to evaluate early progenitor response and 9 weeks post-surgery to determine late healing process by microCT (μCT) and histology. Defect area plus 30% of the defect size on both sides were analyzed when performing histological analysis. For the section analysis automated cell counting was performed^3^. We evaluated the SMA9 cells (red cells), Col2.3GFP osteoblasts (green cells), and SMA9/Col2.3GFP double positive osteoblasts (red and green = yellow) derived from αSMA progenitors. For the μCT analysis defect femurs were scanned at 12 μm voxel size, at 55 kV, 145 µA, 300 ms integration and bone volume determined in the area of the defect. Segmentation of bone was performed with a constrained Gaussian filter to reduce noise, applying standardized threshold (290).

To determine critical size defect healing ability with inhibition of PDGFRβ early in a healing process, 2 days before surgery till 2 days post-surgery animals were treated with either Su16f (10 mg/kg/day in 10% DMSO, oral gavage, Tocris) or Imatinib (50 mg/ml/day in 2% DMSO, 30% PEG250, 2% Tween 80, i.p., Sellechem). Collagen adsorptive scaffold was treated with 5 μg of BMP2 or PBS on a day of surgery. Femurs were evaluated 9 WPD by μCT as described in methods. Critical size defect healing ability with deletion of PDGFRβ in osteoprogenitors was determined in αSMACreERT2/PDGFRβ^fl/fl^ mice (tamoxifen injections on -3/0/3 days of a defect surgery). 5 μg BMP2 was loaded on a scaffold using Cre^−^ and Cre^+^ mice.

### Statistical analysis

All experiments were performed to include at least three biological replicates. Number of animals used for each experiment is listed in figure legends. All data are presented as mean value ± standard error mean (s.e.m.) unless stated otherwise. GraphPad Prism 6 software was used to perform statistical analysis, and depending on data set, two-sided *t*-test, one-way or two-way ANOVA with appropriate post hoc test was used for data analysis. Additionally two-sided *t*-test was used to compare difference between the treated groups of same growth factor treatment. *P* < 0.05 was set as a statistically significant difference between tested groups.

## Supplementary information

Supplemental material

## Data Availability

The data that support the findings of this study are available from the corresponding author on request.
