# Supplementary material for: PDGF inhibits BMP2-induced bone healing
Source: NPJ Regen Med. 2023 Jan 11;8:3. doi: 10.1038/s41536-023-00276-5 (PMC9834334; doi:10.1038/s41536-023-00276-5)
Supplement: Supplementary file 1 — Supplemental material [file 41536_2023_276_MOESM1_ESM.pdf]

Supplemental Table 1. Antibodies used for flow cytometry and western blots.

Antibodies	Catalog number	Producer	Dillution
CD45 violet Fluor 450, Clone: 30-F11	48-0451-82	eBioscience	1:400
Ter119 eFluor 450, Clone: TER-119	48-5921-82	eBioscience	1:400
CD31 eFluor 450, Clone: 390	48-0311-82	eBioscience	1:200
CD140a PE-Cy7 or APC, Clone: APA5	25-1401-82 or 17-1401-81	eBioscience	1:400
CD140b APC or eFluor780, Clone: APB5	17-1402-82 or 47-1402-82	eBioscience	1:100
Phospho-p44/42 MAPK (Erk1/2) (Thr202/Tyr204)	#9101	Cell Signaling	1:1000
Phospho-Akt (Ser473)	#9271	Cell Signaling	1:1000
Anti-rabbit IgG, HRP-linked Antibody	#7074	Cell Signaling	1:2000
GAPDH	sc-25778	Santa Cruz	1:1000
p44/42 MAPK (Erk1/2) Antibody	#9102	Cell Signaling	1:1000
Akt (pan) (40D4) Mouse mAb	#2920	Cell Signaling	1:200
Anti-mouse IgG, HRP-linked Antibody	12.19.16	KPL	1:5000
Osterix	ab209484	Abcam	1:200

Supplemental Table 2. Primers used for gene analysis and recombination and TaqMan probes

Gene	Forward (5'-3')	Reverse (5'-3')
Dlx5	GCC CCTA CCA CCA GTA CG	TCA CCA TCC TCA CCT CTG G
Noggin	CAC TAT CTA CAC ATC CGC CCA G	AGC GTC TCG TTC AGA TCC TTC T
Id1	CTC TAC GAC ATG AAC GGC TGT	TGC TCA CCT TGC GGT TCT G
Pdgfrb	AGG ACA ACC GTA CCT TGG GTG ACT	CAG TTC TGA CAC GTA CCG GGT CTC
***Pdgfrb* recombination**	GGA AAA GCA GGT TTG TGC	CCA GTT AGT CCA CTT ATG TTG
**TaqMan probes**		
Alp	Mm01187117_m1	
Ibsp	Mm00492555_m1	
Bglap	Mm03413826_mH	
Pdgfa	Mm00440701_m1	
Gapdh	Mm99999915_g1	

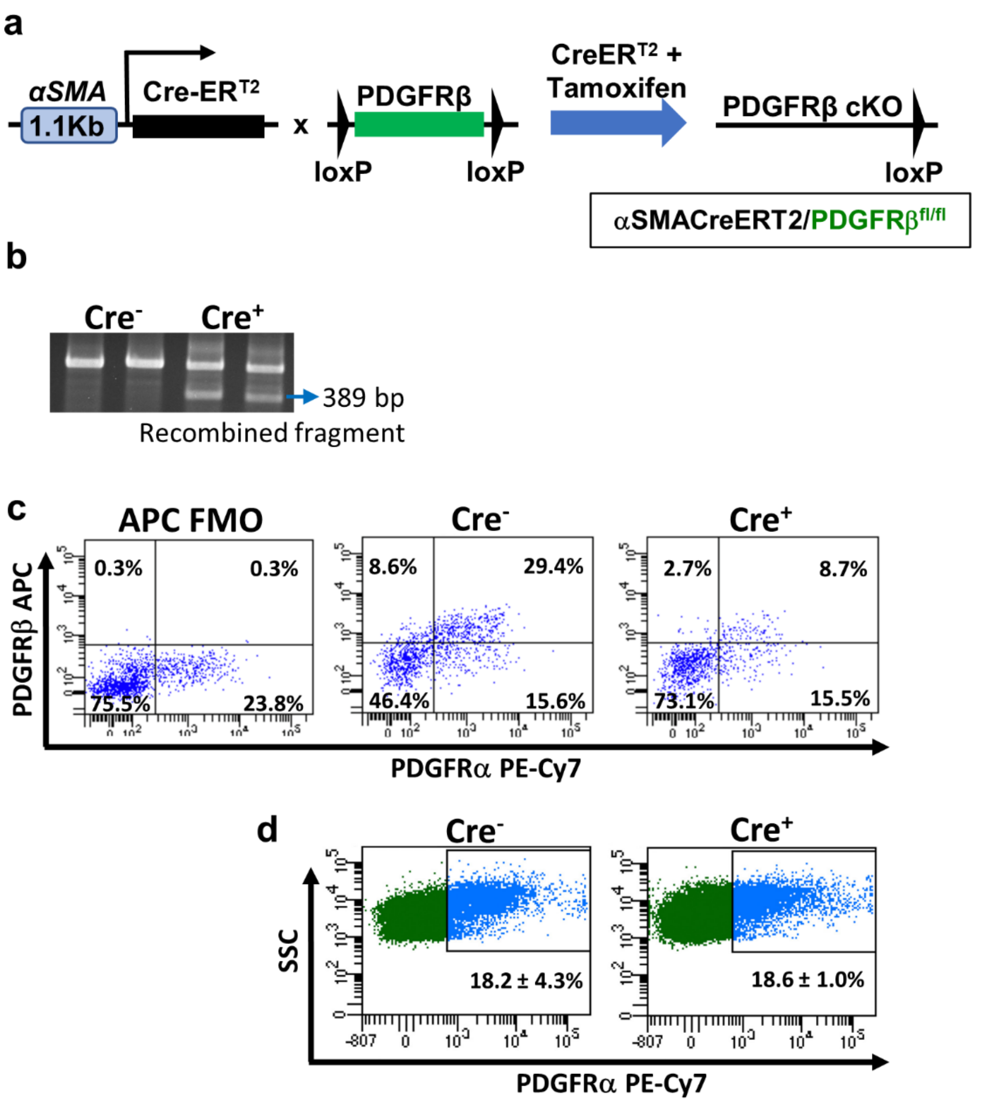

Supplemental figure 1. PDGFRβ recombination efficiency. a. αSMACreERT2 transgenic mice were crossed with a PDGFRβ floxed mice to get αSMACreERT2/PDGFRβ$^{fl/fl}$ mice where upon tamoxifen-induced Cre recombination PDGFRβ is deleted in αSMA cells. b. Cre-induced recombination was validated by DNA recombination in tibia fracture after tamoxifen injection on the day of fracture, 2 and 4 days after in male/female mice. Each lane represents an individual mouse. c. Validation of Cre-induced recombination in fracture of αSMACreERT2/PDGFRβ$^{fl/fl}$ mice evaluating protein expression by flow cytometry 11 DPF in female mice. d. PDGFRα cell proportion within (CD45/Ter119/CD31)$^-$ population is not changed with PDGFRβ deletion compared to Cre$^-$ control on 5 DPF, Cre$^-$ n=3, Cre$^+$ n=2.

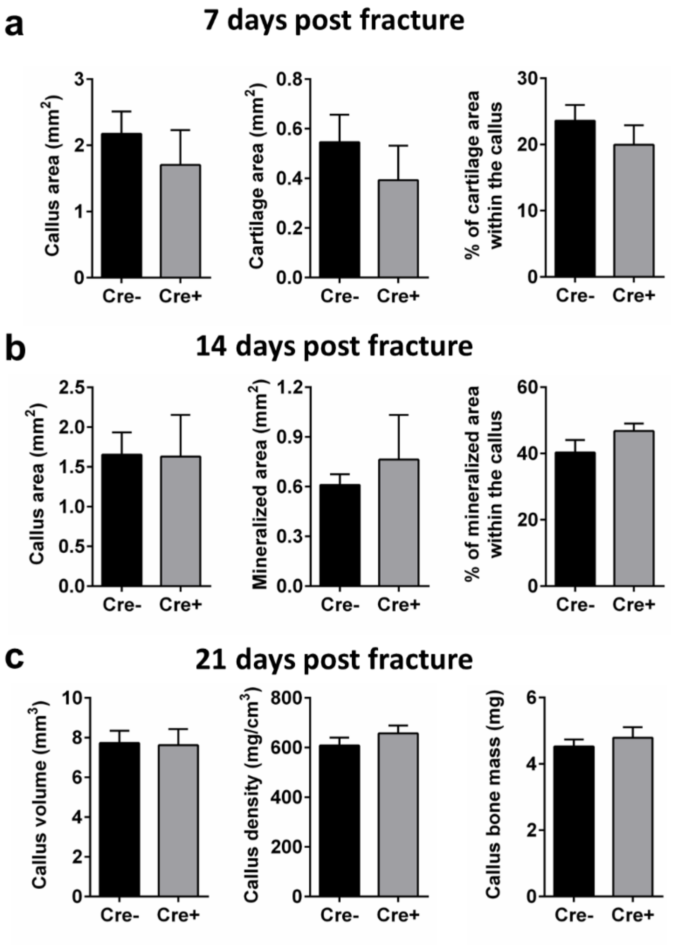

Supplemental figure 2. Fracture healing in αSMACreERT2/PDGFRβ$^{fl/fl}$ female mice. PDGFRβ deletion in αSMA osteoprogenitors was induced injecting tamoxifen on a day of a femur fracture and 2 DPF. a. Callus and cartilage area were determined 7 DPF (n=6), and b. callus, and mineralized area on 14 DPF (n=7). PDGFRβ deletion in αSMA osteoprogenitors did not lead to changes in callus volume and bone mass 21 DPF evaluated by μCT (n=11). Values are expressed as mean + s.e.m. Statistical test: an unpaired two-tailed Students t-test.

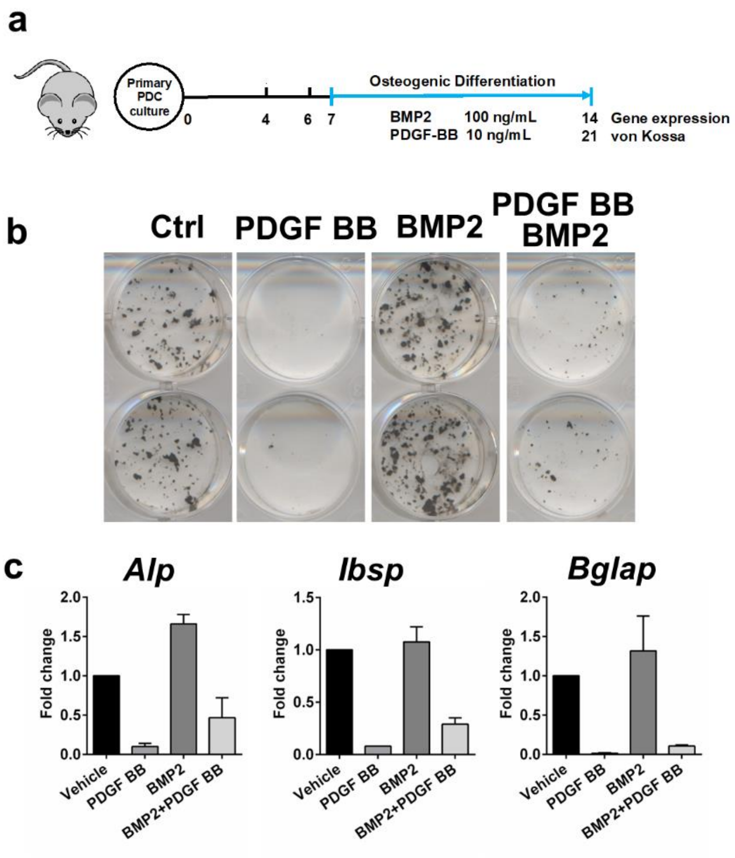

Supplemental figure 3. Effect of PDGF BB on BMP2 osteogenesis. a. Experimental design for induction of periosteal cell osteogenesis b. Von Kossa staining of periosteal cell culture on day 21. c. Gene expression analysis of osteogenesis markers (*Alp*, *Ibsp*, *Bglap*) of periosteal cells was performed on day 14. Values are expressed as mean + s.e.m. n=2. Statistical test: One-way ANOVA with Bonferroni's post hoc test.

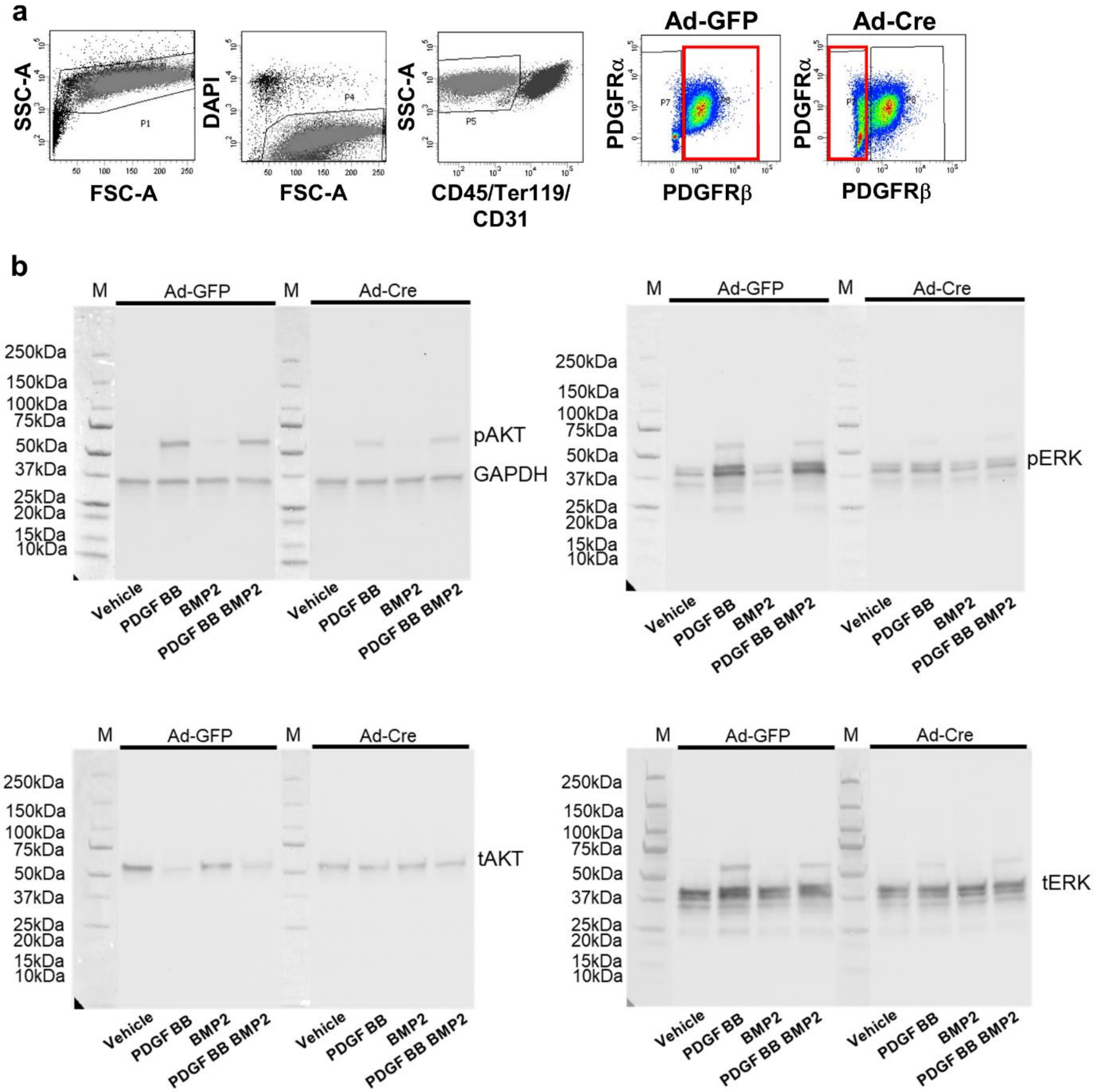

Supplemental figure 4. Protein analysis for sorted PDGFRβ⁻ and PDGFRβ⁺ cells. a. A gating strategy for sorting Ad-GFP PDGFRβ$^+$ cells and Ad-Cre PDGFRβ$^-$ cells after transduction. b. Whole blots after immunoblotting with pAKT, GAPDH, pERK1/2, tAKT and tERK. M – protein ladder. Colorimetric image of a marker is stitched into the original blot to visualize protein size.

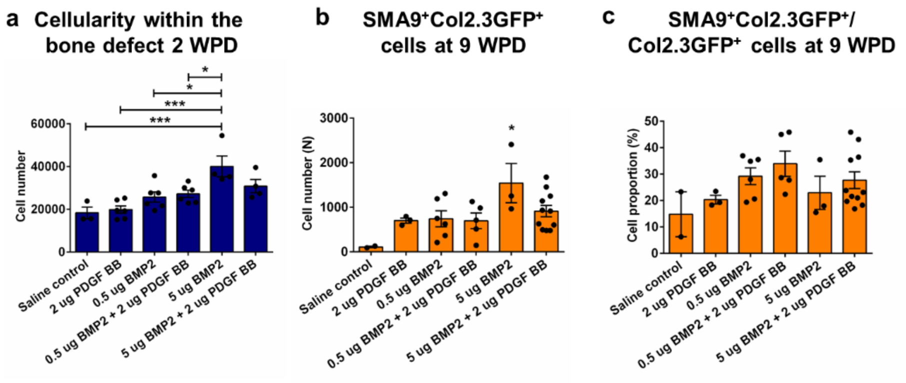

Supplemental figure 5. Histological analysis of femoral defects. a. Femoral defects performed on SMA9/Col2.3GFP male mice and total cell number within the defect area were evaluated 2 WPD. Results are expressed as mean + s.e.m. $*p<0.05$, $*p<0.001$, n=3-6. b. Histological evaluation of dual expressing SMA9^{+}Col2.3GFP^{+} cells within defect area and c. proportion of SMA9^{+}Col2.3GFP^{+} over Col2.3GFP^{+} cells 9 WPD surgery. Results are expressed as mean + s.e.m. $*p<0.05$ compared to saline control, n=5-10 (saline control, n=2) . Statistical test: One-way ANOVA with Tukey's post hoc test.
